# Poor reproducibility of PIRADS score in two multiparametric MRIs before biopsy in men with elevated PSA

**DOI:** 10.1007/s00345-018-2252-4

**Published:** 2018-03-05

**Authors:** Stig Müller, Gunder Lilleaasen, Tor Erik Sand, Lars Løfsgaard, Manuela Estop-Garanto, Dan Helgø, Peter Sund, Vegard Mygland

**Affiliations:** 1 Department of Urology, Akershus University Hospital, Lørenskog, Norway; 2 Institute of Clinical Medicine, University of Oslo, Oslo, Norway

**Keywords:** Prostate cancer, Multiparametric MRI, Biparametric MRI, PIRADS score

## Abstract

**Purpose:**

Since January 2015, all men referred to urologists in Norway due to elevated PSA or other suspicion of prostate cancer underwent multiparametric MRI (mpMRI) before prostate biopsy. At our hospital, patients and the initial MRI were assessed by an urologist and if deemed necessary, patients were referred to another institution for MR/US fusion biopsies. Before MR/US biopsy, patients underwent a second mpMRI. Since we noticed disagreement of these two mpMRIs before biopsy, we retrospectively assessed the level of agreement between the two mpMRIs from the two institutions.

**Methods:**

During the first 6 months of 2015, 292 patients were referred to our outpatient clinic. We referred 126 patients of these to the other institution for MR/US fusion biopsy. The 2 mpMRIs were performed within 4 weeks. We analyzed MR reports and schematics for number of lesions and highest PIRADS score per side of the prostate and histological result of the biopsies. Bland–Altman’s plot was used to compare the level of agreement between the two mpMRIs of the same patient before biopsy.

**Results:**

There was a poor level of agreement between the two mpMRIs and a statistically significant difference in PIRADS scores. Regression analysis showed that there was no proportional or systematic bias.

**Conclusion:**

In unselected patients with elevated PSA, there seems to be a significant variation of mpMRI results across institutions. The PIRADS scoring system needs to be validated with regards to MR equipment, mpMRI protocols and inter-reader variability of radiologists.

## Introduction

Over the past decade, multiparametric magnetic resonance imaging (MR) is increasingly utilized in the diagnostic pathway for prostate cancer. Traditionally, patients with elevated PSA or other suspicion of prostate cancer are investigated by systematic or “random” biopsies guided by transrectal ultrasound (TRUS). The advent of MR as a supplemental imaging modality has led to the implementation of MR-based diagnostic pathways along with the development of equipment that facilitates MR/TRUS fusion-guided biopsies. This novel approach to prostate cancer diagnostics has made targeted biopsies possible, whereas systematic TRUS-guided biopsies often missed lesions in the prostate and has been adopted in many centers. Initially, the use of MR in patients with prior negative systematic biopsies and persisting suspicion of prostate cancer increased cancer detection rates and the use of MR in this setting is strongly recommended in the latest European guidelines [1]. Furthermore, MR-based pathways have been introduced in the primary diagnostic pathway, i.e., before systematic TRUS biopsies. Some randomized trials have confirmed superior cancer detection rates [2, 3], while others have found no benefit [4]. The recently published PROMIS trial advocates the use of MR as a primary triage test before prostate biopsy [5]. However, since MR was increasingly used in preoperative planning before radical prostatectomy and prostate biopsies hamper the interpretation of MR 4–6 weeks after biopsy, a national guideline in Norway was issued in 2014 that all patients with elevated PSA or other suspicion of prostate cancer potentially eligible for radical treatment should undergo multiparametric MR before biopsy as of January 2015.

## Methods

Since January 2015, all patients referred to our outpatient clinic due to elevated PSA and/or other suspicion of prostate cancer eligible for potential radical treatment underwent multiparametric MRI at our institution before the consultation at the outpatient clinic. In the first 6 months of 2015, equipment for MR/TRUS fusion biopsy was not available at our hospital due to lack of funding. Therefore, patients had to be referred to another institution when MR/TRUS fusion biopsy was deemed necessary. The initial mpMRI was classified by the PIRADS scoring system. In addition, the reporting radiologist produced a standardized schematic of the prostate where lesions with the assigned PIRADS score were marked in the prostate. During the first consultation in our outpatient clinic, it was assessed whether the lesion was accessible for systematic and/or cognitive targeted biopsies guided by TRUS (BK Ultrasound, Herlev, Denmark). If yes, TRUS-guided biopsies were performed (MR1 pathway).

Patients with MR lesions that were assessed to be difficult to be reliably accessed by systematic or cognitive biopsy were referred to the other institution. There were no clear selection criteria for referral to MR/TRUS fusion biopsy. The consulting urologist at the first patient visit to the outpatient clinic assessed whether systematic and/or cognitive biopsies were sufficient to clarify the suspicion of prostate cancer. If not, the patient was referred to MR/TRUS fusion biopsy. At the other institution, a new mpMRI was performed before prostate biopsy for convenience, since MR images were directly copied to the fusion biopsy equipment. A MR/TRUS computer-assisted image fusion system with real-time 3D tracking technology (UroStation; Koelis, Grenoble, France) was used for all patients in this pathway (MR2 pathway).

There were apparent, diverging findings in the two MRs in this pathway. Therefore, we retrospectively compared the reports of the two MRs for quality assurance. The study was approved by the Institutional Review Board. Both MRs were performed within 4 weeks without any intervention to the prostate between the exams. The written MR report and the schematic drawn by the reporting radiologist were used to record the highest PIRADS score per left or right side of the prostate. Midline lesions were recorded as left-side lesions in the analysis. The highest PIRADS score per prostate side in the two MRs of the same prostate was then compared.

Both centers used a biparametric MR screening protocol consisting of T2-weighted and diffusion-weighted images without endorectal coil. The reporting radiologist did not disclose in the report whether version 1 or 2 of the PIRADS scoring system was used. Version 2 was published and implemented during 2015 [6].

The histological results of the biopsies were analyzed with regard to cancer detection rate, stratified by highest PIRADS score in the MR. The biopsies from both pathways were processed and evaluated by the department of pathology at our institution.

### Statistical analysis

The difference of the highest PIRADS score of each prostate side where a lesion was reported in one or both MRs was compared with a one-way *t* test against 0. Regression analysis was performed to test for systematic bias between the two PIRADS scores of corresponding prostate sides. SPSS^®^ Statistics version 22 (IBM^®^, Armonk, NY, USA) was used for statistical analyses. A Bland–Altman plot was used to assess the level of agreement between the highest PIRADS score per corresponding prostate side in the two MRs [7].

## Results

**Fig. 1:**
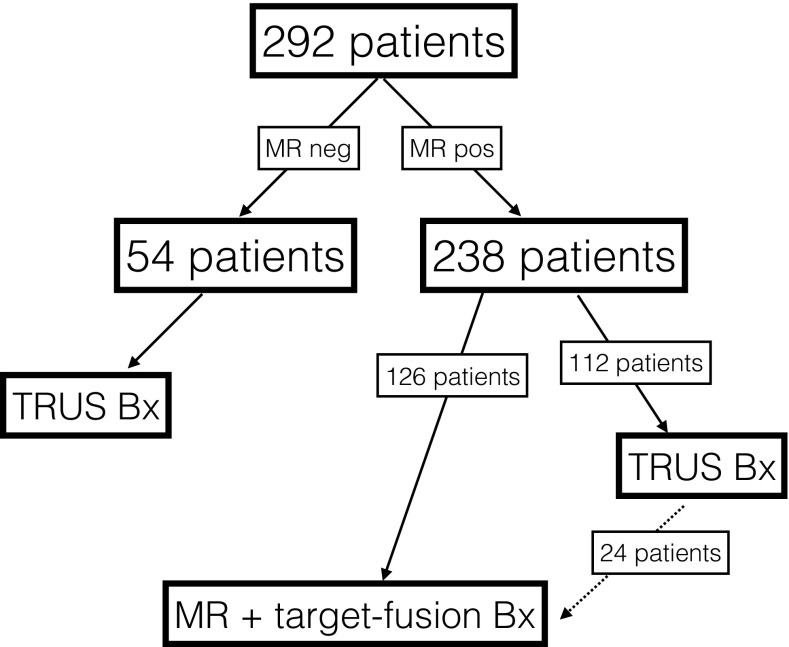
Patients pathways during the study period. 24 patients were referred to institution 2 after TRUS biopsy at institution 1. These were the notes included in the analysis. Only the 126 patients that were referred to institution 2 before biopsy were included in the analysis

During the first 6 months of 2015, 292 patients were referred to our outpatient clinic and underwent mpMRI before the first visit to the outpatient clinic. The distribution of the different pathways after the initial MR is shown in Fig. 1. Of the 238 patients with positive MR findings, 112 patients underwent systematic biopsies at our outpatient clinic (MR1 pathway). In 126 patients, a US/MR fusion was deemed necessary and a subsequent MR was performed before MR/TRUS fusion biopsy (MR2 pathway). The clinical patient characteristics are shown in Table 1. Patients in the MR1 pathway had significantly higher PSA (*p* < 0.01) and a higher proportion had palpable tumors.

**Table 1:** Clinical patients’ characteristics

	MR1	MR2
Age		
Median (range)	66 (47–84)	67 (46–81)
PSA		
ng/ml, mean (range)	13 (0.8–87)	8.6 (0.9–37)*
DRE		
Clinical stage		
cT1c	51%	68%
cT2	39%	32%
cT3	10%	0%
Prostate volume		
ml, mean (range)	52 (17–193)	54 (14–150)

*MR1* MR at institution 1 + TRUS-guided biopsy; *MR2* MR at institution 1, referred to institution 2 for MR/TRUS fusion biopsy based on MR from institution 2

*p < 0.01

**Fig. 2:**
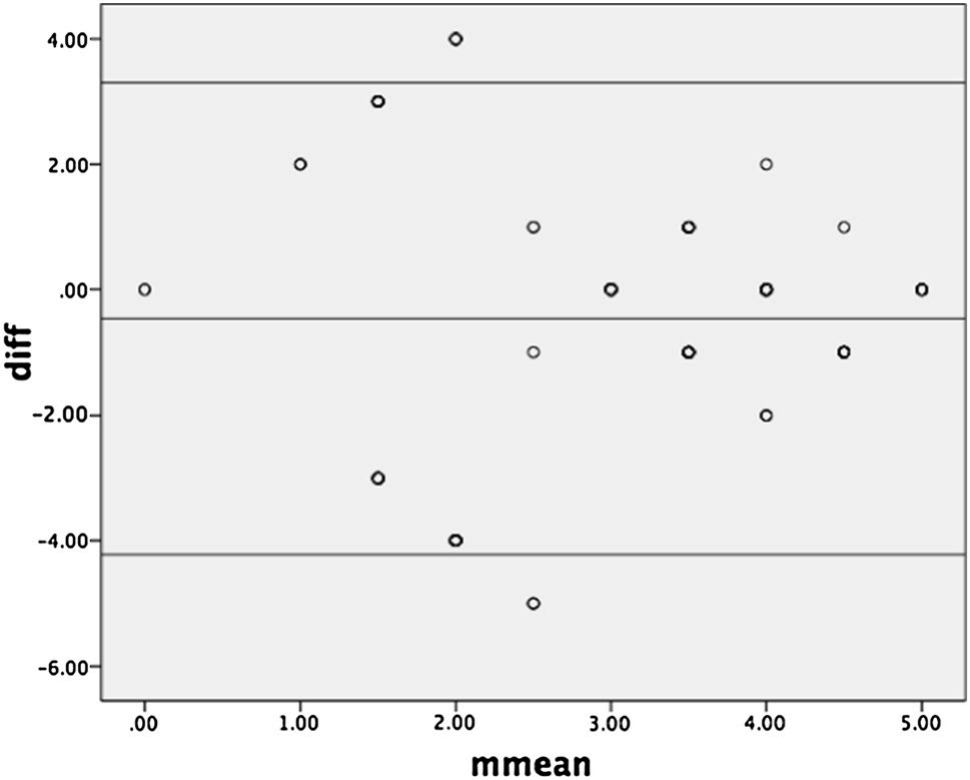
Bland–Altman *plot. mmean* means PIRADS score per prostate side/patient; *diff* difference of PIRADS score in each reported lesion per prostate side/patient

In the 126 patients that were referred to MR/TRUS fusion biopsy, two MRs were available for comparative analysis. The review of the report and schematics revealed 216 lesions for comparison with regard to highest PIRADS score per prostate side/patient. The statistical analysis of the difference of the highest PIRADS scores in corresponding prostate sides of the patients showed a significant difference from zero (*p* < 0.001). Regression analysis revealed no correlation, i.e., no systematic bias. The Bland–Altman plot showed a very poor level of agreement (Fig. 2).

The histological cancer detection rate, stratified by PIRADS score are shown in Table 2.

**Table 2:** Cancer detection stratified by maximum PIRADS score in MR

	MR1			MR2		
	*n*	Cancer detection [*n* (%)]	No biopsy [*n* (%)]	*n*	Cancer detection (*n* (%)]	No biopsy [*n* (%)]
PIRADS 3	33	7 (32)	11 (33)	28	4 (33)	16 (57)
PIRADS 4	44	31(74)	2 (5)	58	36 (81)	14 (24)
PIRADS 5	33	30 (91)	0 (0)	32	30 (100)	2 (6)

Cancer detection percentage is shown as any cancer detected in patients that had a biopsy

*MR1* MR at institution 1 + TRUS-guided biopsy; *MR2* MR at institution 1, referred to MR at institution 2, MR/TRUS fusion biopsy based on MR from institution 2

## Discussion

The main finding of this study is the poor level of agreement between two MRs of the same prostate within 4 weeks without intercurrent prostate intervention. The results of the two MRs expressed by the highest PIRADS score per side of the prostate were significantly different. In addition, there was no systematic bias, i.e., neither MR1 nor MR2 scored lesions systematically higher or lower. To our knowledge, this is the first study that addresses the reproducibility of mpMRI in patients in a diagnostic pathway for prostate cancer. The results from the two MRs were assessed from a clinical viewpoint namely as highest PIRADS score per prostate side, since the PIRADS score is used to decide on the indication for prostate biopsies [8]. It is important to note that our analysis is retrospective and there were no clear selection criteria for the referral to MR/TRUS fusion biopsy. Thus, the patients in the two pathways are different with regard to PSA level and proportion of palpable tumors. However, our cohort of consecutive patients in a “MR-first” diagnostic pathway represents real-life clinical practice. The retrospective analysis of all patients during the first 6 months of this “MR first” pathway shows that patients with higher PIRADS scores are more likely to undergo prostate biopsy (Table 2). Among the patients where the initial MR showed no suspicious lesions (negative MR), only 50% had a prostate biopsy at all. A negative MR seems to dilute the indication for prostate biopsy, even some of these patients have significant cancer. Of the 27 patients with negative MR that had a prostate biopsy, 8 had cancer (5 Gleason 6, 3 Gleason ≥ 7).

There are several limitations regarding our analysis. The two MRs were assessed from a clinical viewpoint, i.e., the reported PIRADS score and localization of lesions with assigned PIRADS score. A number of factors play a role in MR imaging: MR protocol, MR manufacturer, field strength and the experience of the reporting radiologist [9]. Both centers in this study have a high volume of prostate mpMRI and experienced radiologists reporting prostate MR. Ideally, the set of two MRs of the same prostate in our analysis should have been systematically reported by at least two radiologists to determine whether the different findings are due to inter-reader variability or other factors. Our analysis does not allow this comparison. In addition, not all patients in both pathways underwent a biopsy, i.e., the diagnostic accuracy, i.e., sensitivity and specificity of the two MRs cannot be evaluated definitely. Radical prostatectomy specimen would facilitate this evaluation [10]. However, the indication for prostate biopsies in a MR-based pathway is dependent on the MR result and the decision on treatment, active surveillance and further follow-up is dependent on the histological outcome of prostate biopsies. Thus, the histological result of the biopsy can be regarded as the endpoint of the diagnostic pathway. The PIRADS scoring system is a tool for stratifying the risk of cancer at biopsy. In this respect, we stratified the cancer detection rate in the both pathways. It is important to note that patients in the MR1 pathway did not undergo targeted biopsies, i.e., there is no confirmation that the MR lesion is hit by a biopsy. In addition, the patients in the two pathways are different, since patients with smaller or ventral lesions probably were selected for MR/TRUS biopsy. Our analysis of the two MRs is limited to patients in the MR2 pathway who had MR/TRUS biopsies based on the second MR, while patients in the MR1 pathway had systematic biopsies based on the initial MR1. Even the patients in the two pathways are different, we have compared the maximal PIRADS score in the prostate and cancer detection in the biopsies stratified by PIRADS score 3,4 or 5 (Table 2). In both pathways, there is an increasing cancer detection in higher PIRADS scores with > 90% cancer in PIRADS 5 findings (MR1: 78% Gleason ≥ 7, MR2: 66% Gleason ≥ 7). Again, this is a retrospective study of consecutive patients from our outpatient clinic and there were no clear criteria for referral to MR2. The patients in the MR1 pathway probably had lesions that were accessible for systematic biopsies/cognitive targeting. However, even with these limitations, both pathways have similar histological outcomes stratified by PIRADS score. Therefore, the poor level of agreement between the initial MR and the second MR is even more concerning.

Both centers used an abbreviated biparametric MR protocol without dynamic contrast enhancement. This approach has a comparable detection rate of prostate cancer compared to multiparametric protocols with intravenous contrast [11, 12] and reduces cost and acquisition time.

The concept of a “MR first” pathway was issued as a national guideline in Norway without evidence to support this strategy. Recently, the PROMIS trial was published as the first major randomized study that advocates MR as a triage test before prostate biopsy [5]. The authors conclude that 27% of primary biopsies can be avoided and up to 18% more cases of clinically significant prostate cancer (csPCa) might be detected compared with the standard pathway of TRUS biopsy. Also, MR used as a first triage test had a sensitivity of 93% and negative predictive value of 89%. Importantly, these values are based on a definition of csPCa of Gleason ≥ 4 + 3 or a maximum core length involvement of 6 mm or more. Although validated in a pathological review of 693 historical prostatectomy specimen treated between 1984 and 2004 [13], this definition is different from the more traditional threshold of Gleason 3 + 4 for csPCa. There is an ongoing debate on the definition of csPCa and regarding the role of mpMRI and its diagnostic accuracy, one has to bear in mind that the definition of csPCa directly affects, e.g., sensitivity and negative predictive value [5, 14].

The evidence for the use of MR in the diagnostic pathway for prostate cancer is generated in expert/tertiary centers. Therefore, quality and reproducibility is of upmost importance. In a recent report from an expert center, PIRADS score distribution and diagnostic accuracy were evaluated across nine radiologists re-evaluating 503 lesions in 409 MRIs. There was a considerable variability in PIRADS score assignment [15]. Hence, reproducibility of prostate MRI seems to be an issue even in expert centers. There is an obvious need for standardization, technical requirements and sufficient training of radiologists to ensure quality and reproducibility [9, 16]. The results of our retrospective analysis of consecutive patients raise concerns over the reproducibility of MR since our data shows a poor level of agreement of two separate MRs of the same patient. The discrepancy between the two MRs requires further radiological investigation.

## Conclusion

This study demonstrates a poor level of agreement between two MRs of the same prostate before biopsy. Although there is a growing body of evidence for the use of MR in prostate cancer diagnostics and active surveillance, reproducibility and quality must be critically appraised in prospective trials. The PIRADS scoring system needs to be validated in a multicentric fashion with regard to MR equipment, mpMRI protocols and inter-reader variability of radiologists.
